# Synthesis of colloidal silica nanofluid and assessment of its impact on interfacial tension (IFT) and wettability for enhanced oil recovery (EOR)

**DOI:** 10.1038/s41598-023-51038-8

**Published:** 2024-01-03

**Authors:** Morteza Mansouri Zadeh, Fatemeh Amiri, Seyednooroldin Hosseni, Reza Ghamarpoor

**Affiliations:** 1 EOR Research Center, Department of Petroleum Engineering, Omidiyeh Branch, Islamic Azad University (IAU), Omidiyeh, Iran; 2 Department of Petroleum Engineering, Masjed-Soleiman Branch, Islamic Azad University, Masjed-Soleiman, Iran; 3 Department of Petroleum Engineering, Faculty of Engineering, University of Garmsar, Garmsar, Iran

**Keywords:** Chemistry, Engineering, Materials science, Nanoscience and technology, Energy science and technology

## Abstract

Ever-increasing global energy demand, from one hand and reduced oil initially in place in oil reservoirs due to production and reduced natural reservoir production capacity, on the other hand, has encouraged researchers to investigate different methods to improve and increase enhanced oil recovery (EOR) from oil reservoirs. One method is to employ nanotechnology in injected water, where nanoparticles could affect interfacial tension (IFT) between water and oil and wettability through properties, including high specific surface area and nanoparticle size. However, a major challenge in using nanoparticles in injected water is the instability of these particles in water, which ultimately reduces the efficiency of EOR. These particles cannot be stabilized through conventional methods at a large scale. In this study, stabilized silica nanoparticles were synthesized in the water phase using sodium silicate and sol–gel processes. The stability of this nanofluid was studied in seawater, and then its effect on IFT and changing wettability was examined. According to the results, seawater containing 40 times diluted nanofluid could obtain 41% reduced IFT and 40% alteration in wettability of carbonate core becoming more water-wet and ultimately 13.7% improved final oil recovery in secondary oil recovery and 8.3% improved final oil recovery in third EOR.

## Introduction

According to population growth and increasing energy demand, optimized production and protection of energy resources, including crude oil, have captured interest^1–4^. Accordingly, various studies and investigations have been conducted and are being conducted. One of the major research areas of upstream industries in Petroleum Engineering is improving production and enhanced recovery from reservoirs^5^.

One method to increase oil recovery is to alter the wettability through water flooding of chemical substances. Even though this method has always been taken into account, it faces a set of problems due to its low efficiency and its impacts. Thereby, the introduction of methods that can enhance and improve the efficiency of this method has always been a challenge. The majority of available waters for flooding are seawater and produced water of oil field after treatment. The efficiency of this process can improve by addition of chemical and organic materials and optimizing the number of existing ions in the water^6–10^.

Selecting a suitable EOR method depends on screening and assessing properties and conditions of a reservoir and economic feasibility study^11^. Over the past 60 years, an important advancement has taken place in the flooding of chemical materials, increasing its potential to be the most important EOR method^12^. Chemical EOR methods are divided into three groups, including polymer injection, surfactant injection, flooding of alkali materials and their compounds. Each chemical substance has a different mechanism in EOR. Surfactant flooding is known as one of the chemical processes of EOR^13^, including alkaline-surfactant (AS) flooding, surfactant-polymer (SP) flooding, alkaline-surfactant-polymer (ASP) flooding and nanoparticle-surfactant (NS) flooding^14^. Using polymer increases the viscosity of flooded water, which increases the efficiency of viscous oil sweep^15^.

Surfactant is mostly used to reduce interfacial tension and alter wettability in order to increase the capillary number and encourage water to move toward production wells^16^. Surfactant adsorption depends on many factors, such as surfactant type, concentration, equivalent weight, ionic strength, pH, salinity, and temperature^17,18^. Along with silicates, phosphates, and carbonates, these factors can lead to surfactant adsorption to rock surfaces and alter wettability^19^.

Nowadays, nanotechnology as first challenge has led to fundamental advances in EOR from oil and gas reservoirs. For instance, the use of smart fluids or nanofluids leads to a change in the wettability of reservoir rocks, which increases the range of drag reduction due to the formation of a new microstructure. In addition, using surfactant containing nanomaterials causes enhanced oil recovery from reservoirs. Obtaining stabilization methods of nanoparticles in the liquid phase for flooding is another challenge in this field^20^.

Kiani et al.^21^ investigated flooding nanofluid synthesized by the nano gamma-alumina. The obtained results indicated that the maximum oil recovery belongs to flooding nano gamma-alumina in salinity equal to 2000 ppm. The use of gamma alumina nanoparticles led to the improvement of oil recovery performance at low salinity and high temperature.

Sun et al.^22^ studied heavy oils with salinity in oil-wet sandstone core samples. The results indicated that the hybrid sample of SiO_2_ + Al_2_O_3_ has the maximum alter in wettability from oil-wet to water-wet from 156 to 21° using 0.1% by weight of nanohybrid SiO_2_ + Al_2_O_3_. The hybrid sample obtained better results than nanomaterials individually.

Nowrouzi et al.^23^ conducted a study on the dependence of nanoparticle performance on properties, such as particle size, concentration, and distribution of nanoparticles. This study indicated that using higher concentrations and smaller TiO_2_ nanoparticles reduces interfacial tension and contact angle. On the other hand, high concentrations and larger sizes of TiO_2_ nanoparticles increase viscosity. Accordingly, the recovery test indicated that nanofluids containing smaller TiO_2_ nanoparticles have higher efficiency.

Wang et al.^24^ studied wettability alteration of low permeability sandstone cores. In this study, they employed SiO_2_-Rhamnolipid nanofluid for enhanced recovery.

In addition to EOR tests, the three-phase contact angle for water/air/rock and saltwater/oil/rock was measured to study the suitable relationship between rock surface and fluid properties. After conducting nine tests of core flooding by water and nanofluid and studying its effect on alteration in wettability, the results indicated that intermediate wettability of rock surface increases by 5.3–6.8% in recovery for water flooding compared to common flooding. Besides, the significant capability of nanofluid in cores with low permeability for enhanced recovery indicates that it is widely applicable in an oil field.

The review of previous researches reveals that due to inherent properties, such as particle size and the high specific area, nanoparticles have attracted a strong interest among scientists and oil companies for EOR applications. In addition, nanoparticles have capability to change the wettability of rock surface from oil-wet toward a water-wet condition so that attached oils on the rock surfaces can be released and produced. Although different nanoparticles for EOR purposes have been applied by several scholars during the last decades^25^ it can be seen that the colloidal form of nano particle in water flooding has not been fully studied. More than that study of stability of nano particle in the form of colloidal for long time with minimum change in the growth of particle size and its impact on the IFT, wettability and recovery changes need to more focused. Therefore, the main focus of this research is to synthesis of colloidal form of nano silica and its stability in the injecting water with different salinity for investigating change of IFT, wettability and oil recovery. The influencing parameters such as colloidal nano silica concentration in the injecting water, IFT and rock wettability that affect the efficiency of a nanoparticle-EOR are investigated and discussed. The rest of this paper is structured in this way: In Section “Materials and methods”, materials and experimental procedure will be described. In Section “Results and discussion” the details way of synthesis colloidal nano particle has described, in section study of results such as stability of nano, IFT, wettability and oil recovery changes have been discussed, Finally, Section “Conclusions”reports the concluding remarks of this research. The concentration and size of these particles and the dispersion and homogeneity are the most important factors affecting their performance. Accordingly, this study aims to synthesize stable silica nanofluid and examine its impact on interfacial tension and wettability for EOR.

## Materials and methods

### Water phase

In the present study, the water phase was employed in the nanoparticle synthesis process and for dilution of injecting water during core flooding and electrical conductivity measurement. The distilled water with the density of 0.998 $\left( {\frac{{{\text{gr}}}}{{{\text{cm}}^{3} }}} \right)$ and viscosity of 1 cp was used for all the tests.

### Electrolyte and oil phase

In the core flooding process, the saltwater solution with sodium chloride with concentration 260,000 ppm, the density of 1.1951 gr/cm^3^, a viscosity of 1.73 cp was employed for initial saturation of reservoir water to simulate the water–oil phase in core rock. Besides, an oil sample obtained from fields in the Southwest of Iran with a density of 0.8432 gr/cm^3^ was employed in the core flooding process. Seawater as electrolyte phase contains various salt ions for core flooding tests. The stability of nanoparticles in the electrolyte phase with different concentrations was examined.

### Nano phase

In order to obtain and synthesize nanofluid, sodium silicate known as water glass was employed as the primary substance, which was obtained from Merck Co. and Na_2_O weight ratio of 5.25–5.8 and density of 1.296–1.396 kg/m^3^. In addition, in order to convert sodium silicate solution to silicic acid, Amberlite IR-120 resin was employed with an approximate diameter of 1 to 2 mm.

### Core rock

Two oil-wet carbonate core rock samples were employed in the flooding test as a porous medium, obtained from one of the reservoirs in Southwest of Iran. Specifications of the core plugs are reported in Table 1. It is worth noting that the specifications and properties of the employed cores are close to each other as they have been cored from the same formation at the same depth.

**Table 1:** Specifications of the carbonate cores.

Core	Depth (m)	Porosity	Air permeability (md)	Absolute water permeability (md)	Density	Irreducible water saturation	Archie classification
1	2507.72	24.16	201.6	123.8	2.81	20.94	I, A/C, VUG
2	2508.07	25.16	347.6	227.3	2.81	17.81	I/II, A/C, VUG

### Core flooding setup

**Figure 1:**
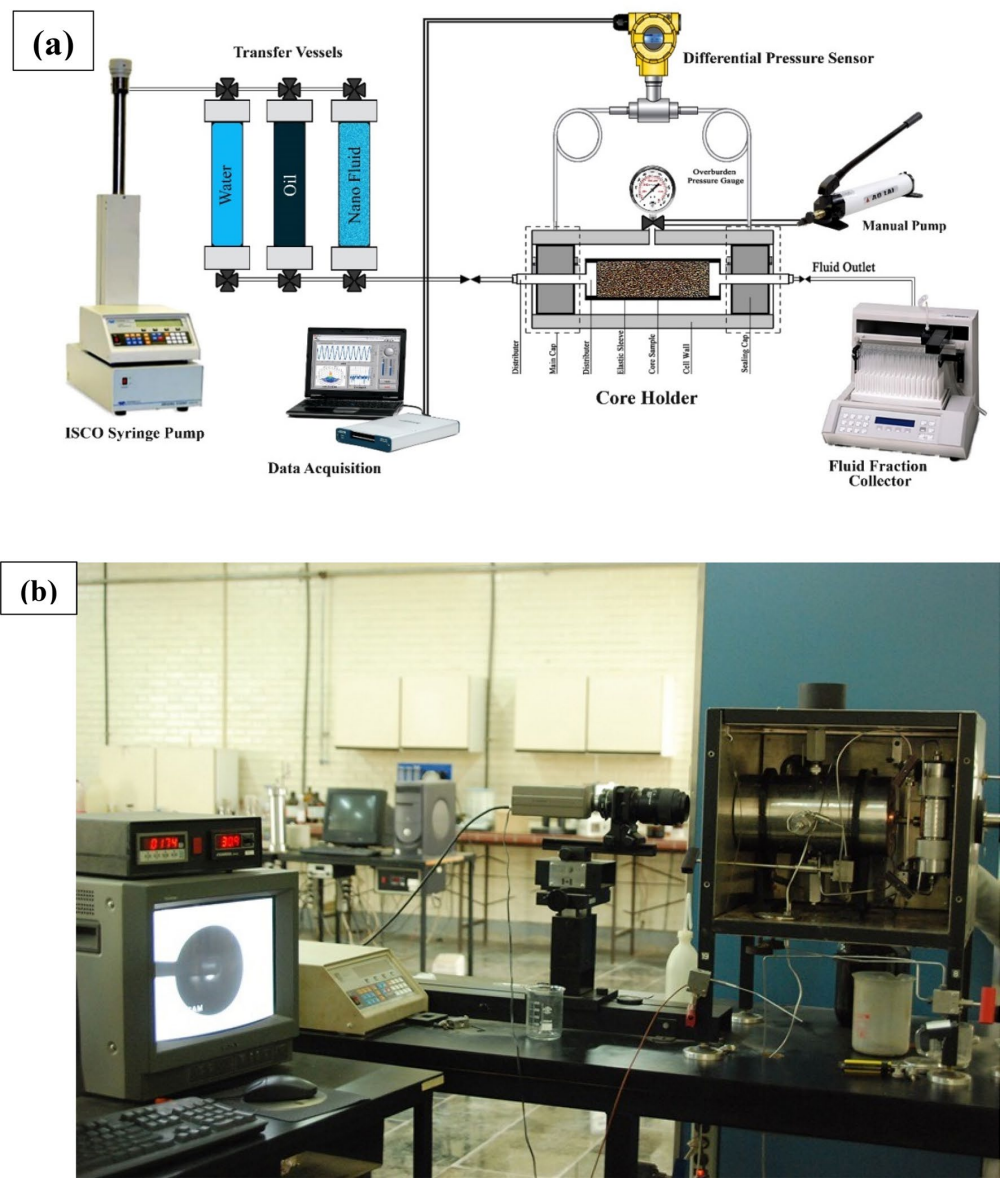
(**a**) Schematic of core flooding device. (**b**) Contact angle measuring device schematic.

A core flooding test was conducted to study effect of nanoparticle solution adsorption in the carbonate core and also its effect in the secondary and tertiary oil recovery. This device consists of five main parts, including high-precision syringe pump (ISCO pump), flooding and production lines, core holder, a production tool based on a data acquisition system and other accessories Illustrated in Fig. 1a.

The injection pump (ISCO pump) employed for water injection to the system was employed under constant flow rate and pressure conditions. Before each test, the core was put in Soxhlet to be washed with toluene for one day and 15 h with methanol. The core was first put inside a rubber cylinder, and then the whole cylinder with the core inside was put inside the holder. Fluid injection flows through the core and not the surrounding since it is covered by a rubber cylinder (plastic or rubber) and under pressure. To fill the brine transfer cylinder, oil, and nanofluid, 2% by weight sodium chloride is required. This amount was added to distilled water. The water transfer cylinder must be completely dried and cleaned before being filled with injection fluid. Transfer cylinder was of piston type such that the side whose fluid is connected to pump must be filled with water and the other side connected to core holder must be filled with flooding fluid. The transfer cylinder output must not be connected to the system until the fluid flows out. At each side of the transfer, the cylinder must lack air, and we must make sure there is no air on both sides.

In the following, the core sample saturated with high saline water, then oil injected with a flow rate of 0.3 cc/min. It must be ensured that the complete replacement of water and oil drains is achieved until irreducible water saturation is reached. The following equation is used to calculate the irreducible water saturation after the displacement test.

1 $$S_{wi} = 1 - \frac{{V_{watre\;produced} }}{{V_{pore} }}$$

where S_wi_ is irreducible water saturation, $V_{watre\;produced}$ is the mount of produced water (cc), and ${V}_{pore}$ is the pore volume of core plug (cc).

The scenario that was planned for this aim has been based on secondary and tertiary oil recovery, such that the reaction between rock and fluid is determined with respect to the pressure and temperature where we can investigate the adsorption as a fundamental parameter. After oil injection, the nanofluid is injected until no oil is produced anymore and we reach the minimum remaining oil saturation (${S}_{or}$) in the plug. The recovery factor (RF) is calculated based on the following equation:

2 $${\text{RF}} = \frac{{1 - S_{wi} - S_{or} }}{{1 - S_{wi} }}$$

The constant injection rate of the saline water was controlled by the ISCO Pump. A constant pressure of 300 psi was considered for the initial saturation of the rock. After the 100% saturation of the rock by the saline water, first, we release the system and choose three different flow rates on the pump so that the pressure fluctuations become constant on a specific pressure with a constant flow rate. Concerning the obtained point with respect to the input variables and by using the Darcy Equation, the core permeability can be calculated.

### Measurement of interfacial tension and wettability

One of the forces affecting the flow of oil in a porous environment is the capillary force, the amount of this force depends on the properties of the reservoir rock, the nature of the surrounding fluid, and the degree of wettability of the rock relative to the fluid. This device measures the property of surface tension which is defined in the case of two liquids adjacent to each other and immiscible. Another application of this device is to obtain the contact angle between rock and fluid. As shown in Fig. 1b, this device provides the possibility of measuring the interfacial surface tension of fluids, both liquid–liquid and liquid–gas, using the pendant drop method. In this method, one of the phases hangs in the form of a drop in the space of the other phase. The geometric characteristics of the drop obtained by photographing it and a computer program determine the interfacial tension between the two phases. In addition, by placing a polished sample of reservoir rock, it is possible to measure the contact angle surface of liquid–liquid or gas–liquid fluids with great accuracy in different conditions of temperature–pressure and composition of fluids by this device. The rock is first fixed in the chamber and inside one of the fixed and the drop or bubble from the other phase is placed in contact with it. The shape of the droplet or bubble after contact with the core is photographed and the contact angle is accurately measured.

### The colloidal nanofluid synthesis process

To prepare the nanofluid solution, sodium silicate has been used as the raw material^26–28^. In the second step, the SiO_2_ and Na_2_O of Na_2_SiO_3_ ratio have been adjusted using cation exchange resin, and its concentration is determined as desired and with the extra water evaporation. The existing Na_2_SiO_3_ becomes the active Silicic acid by adjusting the specific condition in terms of the column length and diameter, the resin capacity determining, and resin and solution temperature while passing through the bed. After nucleation and stabilization, the next step is to dilute unstable silicic acid to silica. In this step, the nucleosynthesis and particle growth are done as desired by adjusting the sodium concentration, the temperature, and the entry rate of the active Silicic acid to the Sodium hydroxide. Afterward, the nanofluid with a high concentration is obtained by discharging the extra water via evaporation under vacuum conditions or the filtration system. Controlling the particles size and the stability of the formed nanofluid depends on the conditions, e.g., the rate of active Silicic acid to the Alkali and its temperature, each of which is optimized in order to achieve the maximum stability (avoiding gelation at the ambient temperature in the long run).

In order to prepare the Silicic acid for the synthesis of the Colloidal silica, the Cationic substitution property of resin was used. For this purpose, cylindrical glass columns with 8 cm in diameter and 50 cm in height were designed. Afterward, a specific volume of the spherical grains of the Amberlite IR-120 cation with 1–2 mm in diameter was placed over one day in the Double Distilled Water. By doing this operation, we aimed to maximize the resin grain expansion volume so that resin volume addition and explosion of the container are prevented at the time of placement inside the cylindrical containers and adding water to them.

Then, the expanded resin grains were poured to the height of 25 cm in the installed column, and then double-distilled water was slowly added to the 3 cm to the upper side of the resin. The glass column was rinsed by using 200 ml Hcl (1 Molar). Afterward, the sodium silicate that is known as glass water prepared by Merck Company with code 1.05621.2500 and ratio of 25.5–28.5% by weight of SiO_2_ and 7.5–8.5% by weight of Na_2_O and density of 1.296–1.396 was used at 20 °C to obtain silicic acid. 37.5 g of this sodium silicate was poured into 200 ml of distilled water and perfectly mixed. Afterward, the obtained solution was rinsed with acid (neutralized with distilled water) and poured into the glass container with cation resin. After 1 h of being inside the column, its valve was opened slowly, and the acidic outlet solution was collected with a pH of about 3.5–4. The solution collection was done until the pH solution reached 4. After collecting the solution, the glass column containing cation resin was rinsed with water several times to be prepared for reuse. The resulting acidic solution was H_2_SiO_3_. According to the structure and free ions in its structure, the resin acts as the effective agent in the ion exchanges ad replacement of Na^+^ and H^+^. The reactions pertinent to the formation of silicic acid are indicated below.

3 $$2{\text{HCL}} \to {\text{Cl}}_{2} + 2{\text{H}}^{ + }$$

4 $${\text{Na}}_{2} {\text{SiO}}_{3} + 2{\text{H}}^{ + } \to {\text{H}}_{2} {\text{SiO}}_{3} + 2{\text{Na}}^{ + }$$

By changing the concentration by weight of sodium silicate, silicic acid with different concentrations was prepared. It is worth noting that controlling the concentration of sodium silicate is of paramount importance to prevent the gelation of the resin inside the column and the preparation of silicic acid with a suitable SiO_2_ concentration. After preparing, 200 cm^3^ of silicic acid, placed inside the glass separatory funnel. Afterward, inside a polymeric cylindrical container with an approximate diameter of 5 cm and height of 10 cm, 1, 2, and 3% glass water solution was prepared in 30 cm^3^ of water (i.e., 1.3, 2.6 and 3.9 gr of glass water in 30 cm^3^ of water). In the following, a set consisting of mixer and heater, iron base and holding clamps, thermometer, and Pyrex container containing silicon oil was manufactured for fast heat transfer with the ability to withstand higher temperatures than 100 °C without being evaporated. When the glass water solution temperature reached 80 to 90 °C, silicic acid was slowly added to the glass water solution through a dropper-shaped separatory funnel with a controlled rate of 0.85 ml/min. By the addition of each drop acid, the required time for the formation of colloidal particles inside the glass water was given, while the volume of solution was kept constant at 30 cm^3^. In other words, the addition rate of acid must be equal to the evaporation rate so that the solution volume remains constant at 30 cm^3^. By addition of acid in such a manner, the formation of colloidal particles inside the solution was more recognizable such that the solution color changed to white gradually. It must be noted that constant control of pH with Litmus is necessary. It is because as pH drops from 11 and its equivalent amount and the pH of the initial sodium silicate solution reaches 7 or 8, the colloidal solution might convert into a gel. Accordingly, the pH of the solution must not drop from 9. Therefore, the pH of the solution was controlled to remain at 9.5–11.5. The reactions pertinent to the formation of colloidal sol of SiO_2_ are indicated below.

5 $$2{\text{H}}_{2} {\text{SiO}}_{3 } \to {\text{H}}_{4} {\text{SiO}}_{4} + {\text{SiO}}_{2}$$

6 $${\text{H}}_{4} {\text{SiO}}_{4} + {\text{NaOH}} \to {\text{NaH}}_{3} {\text{SiO}}_{4} + {\text{H}}_{2} {\text{O}}$$

7 $${\text{NaH}}_{3} {\text{SiO}}_{4} + {\text{Na}}_{2} {\text{SiO}}_{3} \to 3{\text{NaOH}} + {\text{SiO}}_{2}$$

8 $$\begin{gathered} {\text{Total}}: \hfill \\ {\text{Na}}_{2} {\text{SiO}}_{3} + 2{\text{H}}_{2} {\text{SiO}}_{3} \to 3{\text{SiO}}_{2} + 2{\text{NaOH}} + {\text{H}}_{2} {\text{O}} \hfill \\ \end{gathered}$$

The concentration of SiO_2_ and pH of colloidal samples were measured using the gravimetric method and pH meter BLD 8252 model. Dynamic optical spectroscopy method was employed using the device model Malvern to measure Zeta Sizer, Nano ZS ZEN 6 3600 potential zetas, and colloidal silica particles. Ultimately, the size and shape of cavities and specific area of particles in the final optimal sample were measured using the BET test, which determines a specific area. In order to assess formed chemical bands accurately, the FTIR test was carried out on the synthesized sample.

## Results and discussion

### Characterization of colloidal nanofluid

**Figure 2:**
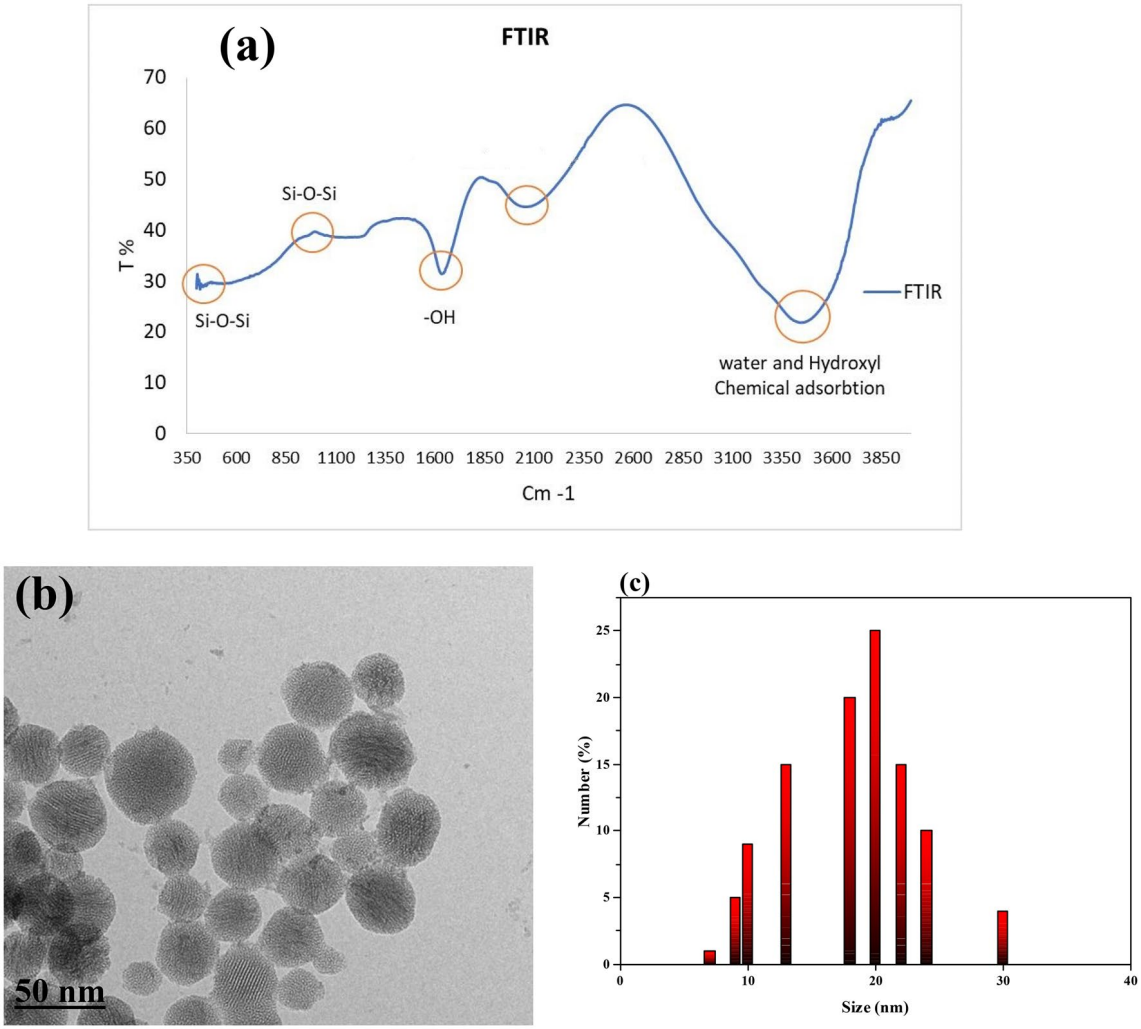
(**a**) FTIR Spectroscopy test (**b**) TEM image and (**c**) Particle size distribution in DI water from synthesized colloidal silica.

According to Fig. 2a, bands between 3450 and 3640 cm^−1^ are formed because of hydroxyl groups. Bands between 1090 and 1100 cm^−1^ belong to the bending vibration of Si–O–Si^29,30^. Besides, bands between 1630 and 1640 cm^−1^ are pertinent to the bending vibration of the –OH group. In addition, bands from 417 to 475 cm^−1^ belong to tensile modes of Si–O–Si^31^. Bands between 2058 and 2095 cm^−1^ belong to the chemical adsorption of water.

The TEM image of the colloidal silica nanoparticles is shown in Fig. 2b. The obtained NPs were amorphous not crystalline in a spherical shape with a diameter of ca. 10–30 nm. The size and the size distribution were further characterized using a nanoparticle size analyzer. The obtained hydrodynamic diameter of the nanoparticles and its distribution is shown in Fig. 2c with an average size of 50 ± 5 nm, which indicated a relatively uniform size and the result was comparable with that from the TEM image.

According to performed analysis, the silica structures are formed in the fluid. Besides, the results of the chemical analysis of the synthesized structure are shown in Table 2, indicating that the concentration of silica in the prepared solution is approximately 24%. The Na^+^ ion in the solution was 3700 ppm, and the structure of particles remained stable because of this amount. In addition, the measuring value of other ions indicates that this synthesized fluid is of high purity. In order to detect nanometric structures in colloidal nanofluid, particle size measurement and zeta potential tests were performed using the DLS test^32^.

**Table 2:** Chemical analysis of synthesized fluid.

Test	Test standard	unit	Result
Chloride	ASTM D512-19	Percentage by weight	0.0162
Sulfate	ICP-OES	Percentage by weight	0.0008
Sodium	ICP-OES	Percentage by weight	0.37
SiO_2_	ISO 1690-1976	Percentage by weight	24.18
potassium	ICP-OES	Percentage by weight	0.008
Na_2_SO_3_	–	Percentage by weight	< 0.001
pH	–	–	9.9

**Figure 3:**
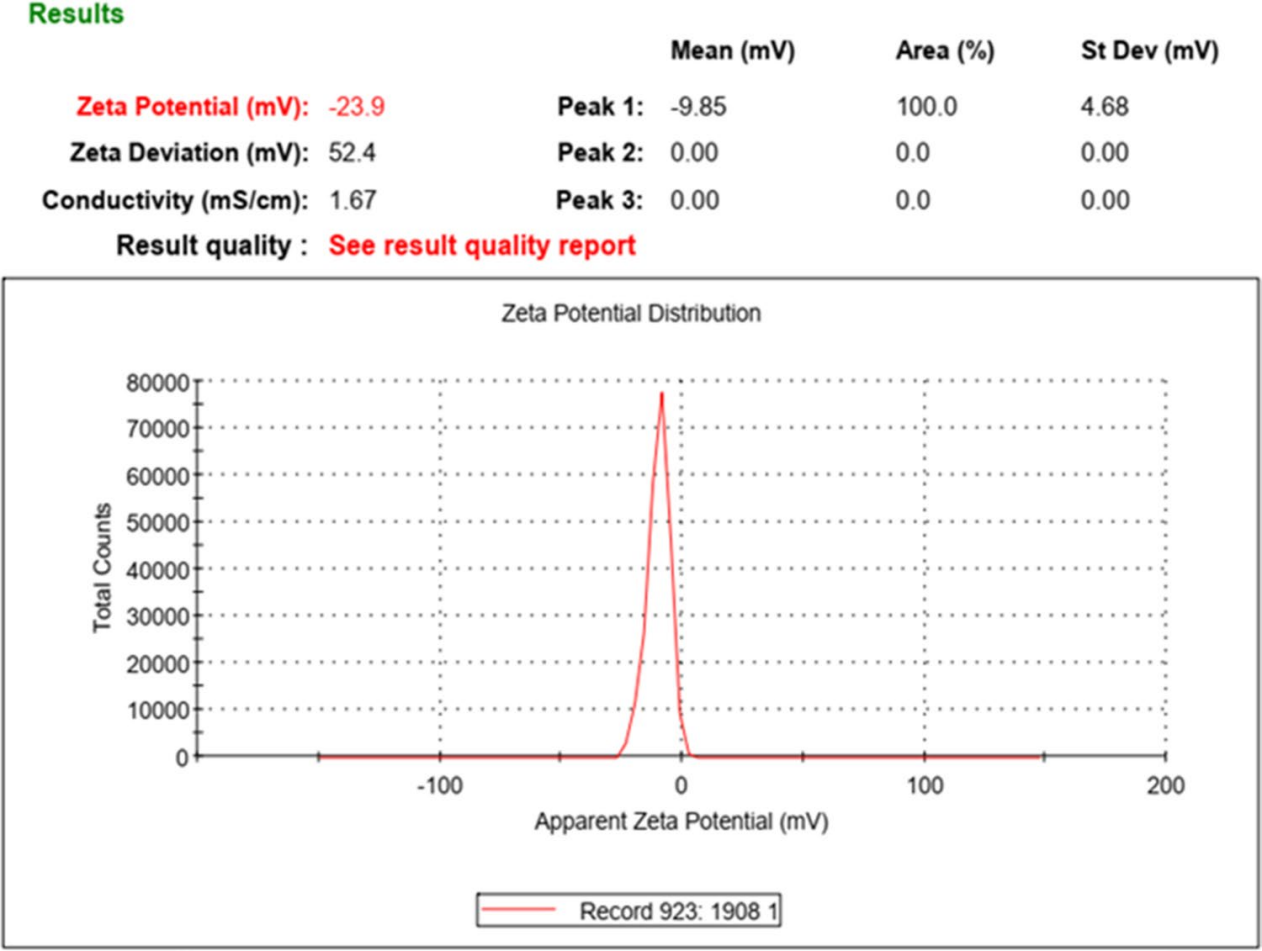
Diagram of synthesized colloidal nano-silica zeta.

**Figure 4:**
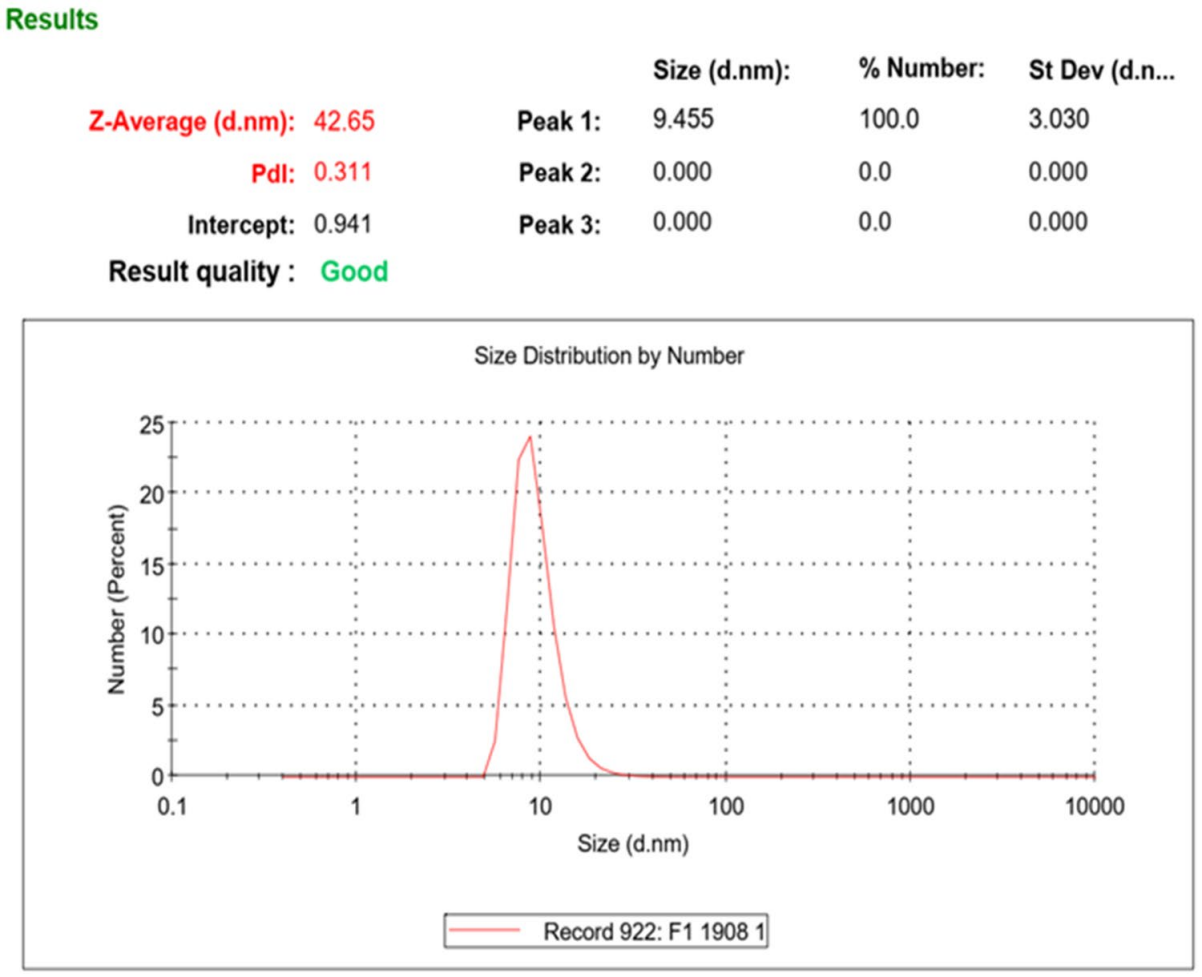
Distribution diagram of other synthesized colloidal nano-silica particles.

According to Fig. 3, the average potential of stabilized nano zeta structure is within the range of -24 mv. It indicates the presence of negative factors and suitable stability by Na^+^ ion according to the high concentration of silica nanoparticles which is approximately 24% in nanofluid structure. According to Fig. 4, the peak of particle sizes is shown at 10 nm. However, due to an insignificant amount of structures with greater sizes, the average size of particles is indicated to be approximately 42 nm. Furthermore, Polydispersity Index (PDI) measures the dispersion of particles individually or multi particles. PDI of < 0.5 indicates that particles are monodisperse and PDI of > 0.5 indicates particles are polydisperse^33,34^.

According to measured values of 0.311 in the DLS test, the formed particles are monodispersed, indicating high dispersion quality of particles. BET is another assessment pertinent to nanometric properties that measure the specific area of nanoparticles and the potential of contact surface formation with the surrounding environment of the particle^35^.

**Figure 5:**
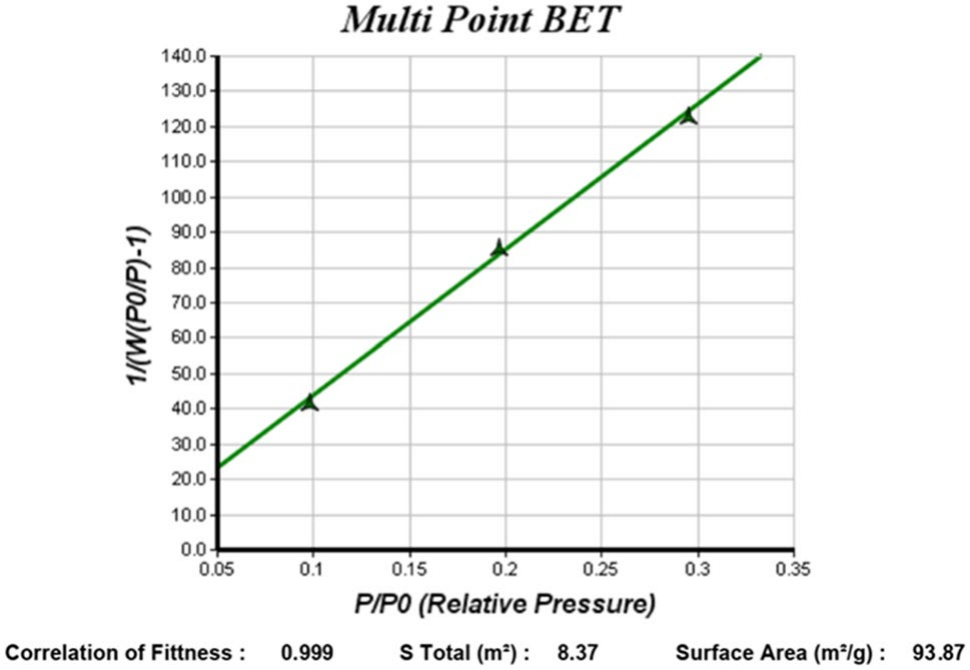
Diagram of specific area analysis of nanoparticles in BET analysis.

**Figure 6:**
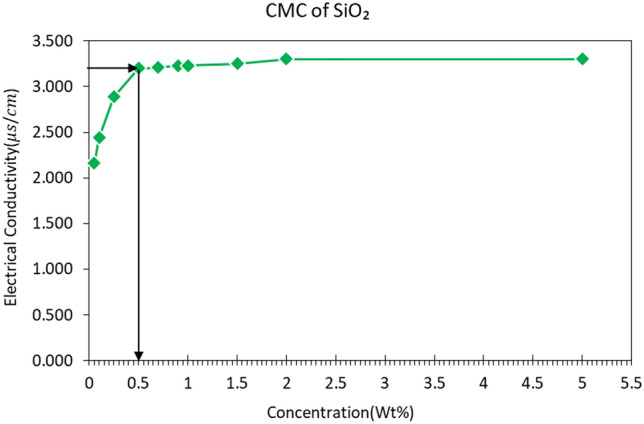
Electrical conductivity at different concentrations of nano-silica solution.

Figure 5 indicates the results obtained by the BET test. According to this test, the specific area is measured to be 93.87 m^2^/g. In order to determine the critical concentration of micelle formation, ten different base concentrations were obtained, and the electrical conductivity of each concentration was measured. The calibration diagram was obtained by measuring electrical conductivity versus Nanofluid concentrations (see Fig. 6). After the micelle structure was formed, the concentration conductivity did not increase at higher concentrations, and it remained constant. Optimal concentration and micelle formation were considered when conductivity is close to zero, and the corresponding concentration was obtained 0.5%.

In order to study optimal concentrations and seawater dilution degrees, nano-silica colloid was examined in terms of stability. Due to the selection of 0.5% by weight as an optimal concentration through conductivity and micelle critical concentration, four scenarios were selected to select flooding fluid according to Table 3.

**Table 3:** Synthesizes scenarios of flooding fluids.

Fluid	Dilution degree	The concentration of silica fluid (%)
Seawater	0	0
Nanofluid 1	40 times	0.5
Nanofluid 2	20 times	0.5
Nanofluid 3	5 times	0.5

### Stability assessment of nanofluid

The prepared colloidal silica nanoparticles were dissolved in hot sodium hydroxide (1 M) and the UV analysis was performed. Pure sodium hydroxide (1 M) was used as blank for the entire studies. After mixing nanofluid and obtaining the desired concentration, nanofluids 1, 2, and 3 that are selected flooding scenarios were observed at three periods of 3, 10, and 21 days through measuring the amount of UV radiation.

**Figure 7:**
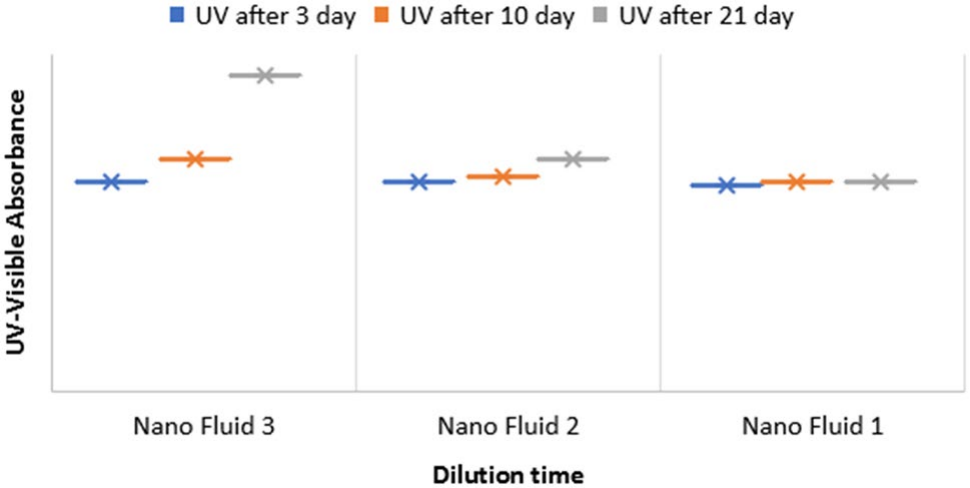
Alterations in UV radiation of three nanofluid over time.

**Figure 8:**
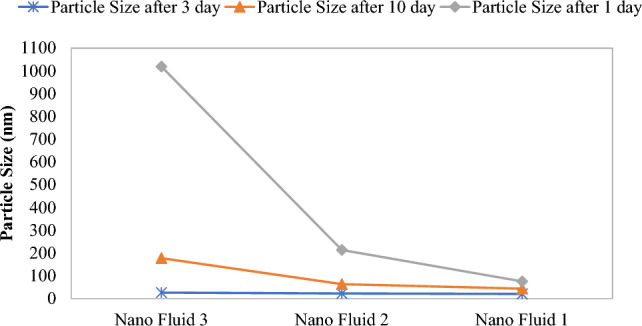
Diagram of alteration in particle sizes in DLS test of fluid containing 0.5% nano colloid in different concentrations seawater at different times at 25 °C.

As indicated in Figs. 7 and 8, nanofluid 1 has insignificant alterations after 21 days, which can be attributed to the low amount of salt ions in seawater which has undergone more dilution, and the concentration of its salts is close to the water with low salinity. Nanofluid 2 has also experienced insignificant alterations for ten days. Nevertheless, it faced significant growth after 21 days. This particle growth can be neglected due to lack of significant growth and also out of nanometric properties. Moreover, the nanofluid 3 faced significant growth in nanoparticle sizes, which can be attributed to the high concentration of salts in seawater, especially ion Na^+^. After ten days, it faced instability and sedimentation. As UV radiation increased, particles accumulated and grew and became out of nanometric form and led to instability of injected fluid.

### Assessment of interfacial tension alterations

**Figure 9:**
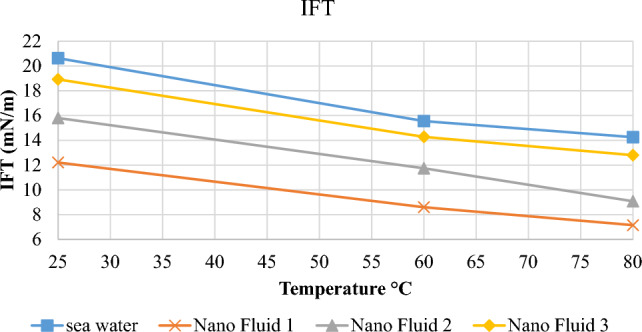
Diagram of alterations in interfacial tension at the presence of seawater, nanofluid 1, nanofluid 2 and nanofluid 3.

In order to assess the effect of colloidal nanoparticles on interfacial tension, the effect of all four synthesized fluids on alterations in interfacial surface tension was assessed by injecting oil at the temperatures of 25, 60, and 80 °C^36,37^. As indicated in Fig. 9, the interfacial tension between seawater and oil at 25 °C was obtained at 20.63 mN/m. In nanofluid 3, the interfacial tension was reduced by 9%. Nanofluid 2 also could alter interfacial tension by 23%. In addition, nanofluid one reduced interfacial tension by 41%, and this reduction was equal to 49% at temperature increase up to 80 °C.

### Assessing alteration in wettability

**Figure 10:**
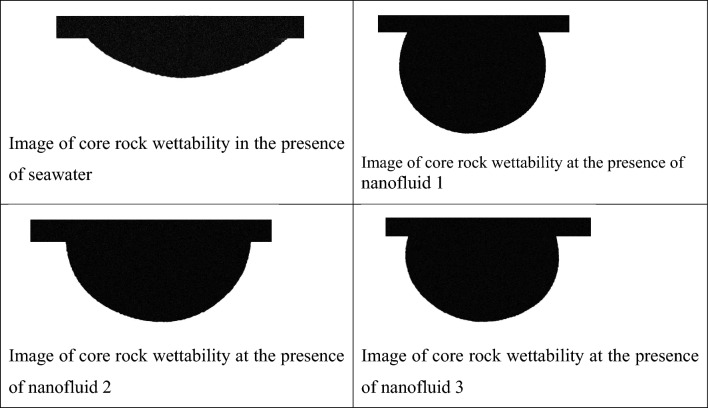
Image of core rock wettability at the presence of seawater, nanofluid 1, 2, and 3.

**Figure 11:**
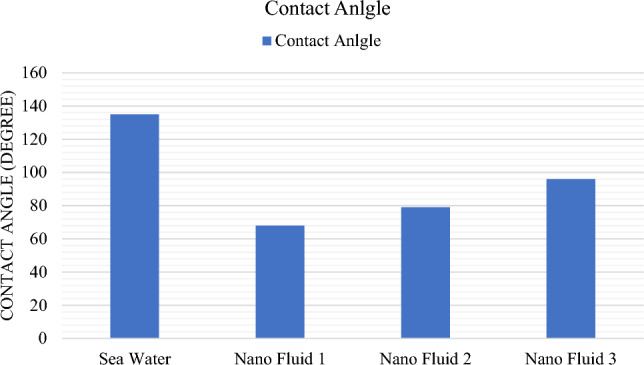
Alterations in contact angle of core rock at the presence of seawater and nanofluids 1, 2, and 3.

One of the influential forces on oil flow in a porous environment is a capillary force that depends on the wettability of rock from fluid and adjacent fluid and the properties of the reservoir rock^38^. In this section, the effect of wettability changes of nanofluids on oil-wet carbonate core rock was investigated. During the production of oil from carbonate reservoir rock, due to its oil-wetting nature and the spread of oil as a wet phase in large pores and adhesion on their surface, part of the oil falls into the trap, which requires the creation of changes in wettability with the help of reducing Interfacial tension can produce this oil^39–42^. As shown in Table 4 and Figs. 10 and 11, the measurement of wettability was tested in the presence of 4 fluids i.e., seawater fluids and nano fluid 1, 2, 3 on carbonate rock samples for 24 h of contact. In the contact of sea water, the wetting angle reached 135° after 24 h. After that, by contacting nanofluid 1, which has less salinity and contain nano particles, this angle changed from 135° to 68° and change its wettability toward hydrophilicity. The effect of creating pressure on the oil film changes the wettability and contact angle of the oil with the rock surface. Also, nanofluid 2 and 3 have also caused a change in wettability angle with 79° and 96°, respectively, and here the most changes in wettability towards hydrophilicity have been caused by nanofluid 1.

**Table 4:** Alterations in contact angle of rock due to contact with seawater and nanofluids 1, 2, and 3.

Fluid title	Secondary contact angle (°)	Contact time (h)
Seawater	135	24
Fluid 1	68	24
Fluid 2	79	24
Fluid 3	96	24

### Assessing the role of nanofluid in oil recovery

**Figure 12:**
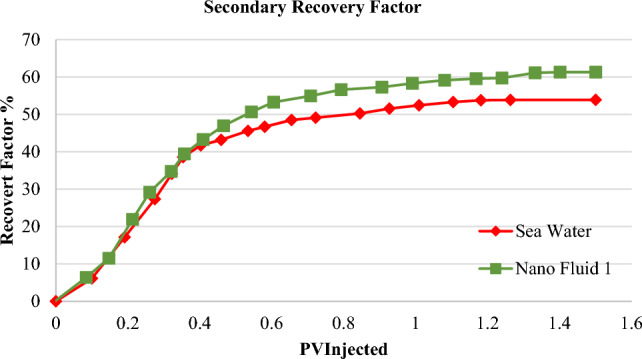
Diagram of secondary oil recovery rate in core one due to flooding by seawater and nanofluid 1.

In order to study the effect of nanofluid injection on enhanced recovery, nanofluid one was selected as the optimal fluid for performing enhanced core flooding tests. For this purpose, two carbonate core rocks with specifications mentioned in Table 1 were selected. The core number 1 was used in the secondary recovery test^36,43–46^. In the first step, Seawater was injected, and the final recovery was 52.88% after injecting 1.5 PV. Similarly, the nanofluid one was selected as an injection fluid in the next case. It obtained a final recovery of 59.30% after injecting 1.5 PV, which improved by 12% compared to injection by seawater. The results of the final recovery are indicated in Table 5 and Fig. 12.

**Table 5:** Summary of obtained results from secondary recovery test.

Flooding fluid	Final secondary recovery (%)	Improvement degree (%)
Seawater	53.88	0
Nanofluid 1	61.30	13.7

**Figure 13:**
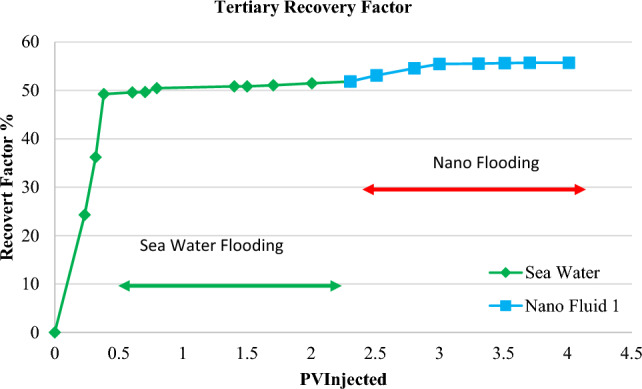
Diagram of secondary oil recovery rate in core two due to flooding by seawater and nanofluid 1.

Core number 2 was employed for the enhanced recovery test. Figure 13 indicates the effect of nanofluid as an enhanced injection. In these tests, 2 PV of seawater was first injected and obtained a constant oil recovery of 51.47%. Then, 2 PV nanofluid one was injected into the core rock. It obtained 55.72% final recovery, which indicates the effect of nanofluid on separation of oil phase because of mechanisms, including alteration in interfacial tension and wettability. Finally, 8.2% increased recovery in core rock was obtained. Table 6 indicates the summary of the core flooding test results for enhanced recovery.

**Table 6:** Summary of obtained results from secondary recovery test.

Flooding fluid	Final secondary recovery (%)	Improvement degree (%)
Seawater	51.47	0
Nanofluid 1	55.72	8.2

### Displacement mechanisms

**Figure 14:**
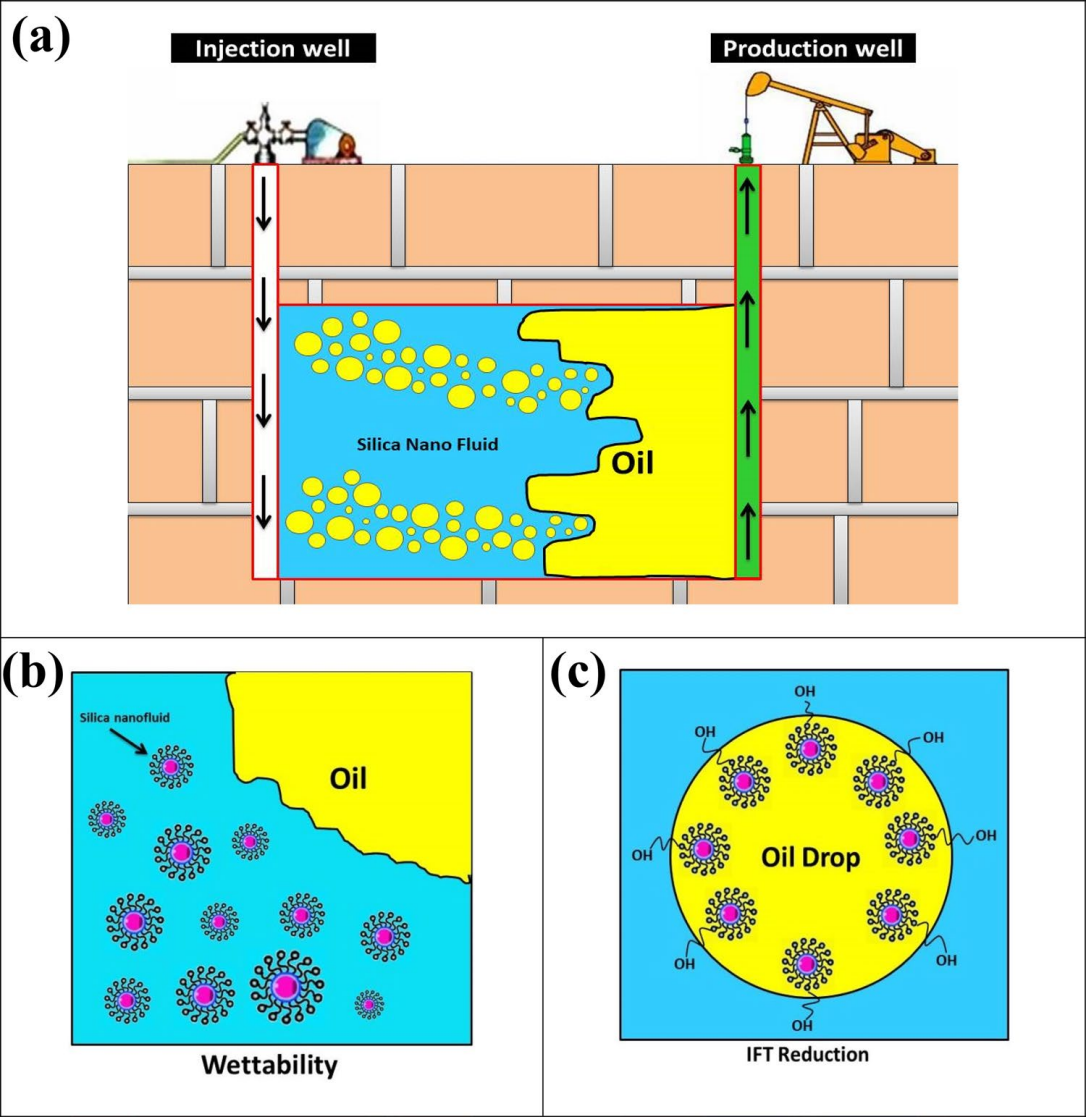
Schematic of mechanisms found after conducting EOR operations using colloidal silica nanoparticles: (**a**) Mobility improvement, (**b**) Wettability alteration, and (**c**) IFT reduction.

In this study, colloidal silica nanoparticles could influence brine viscosity and favorably modify mobility ratio and resulted in strong macroscopic efficiency (see Fig. 14a). Hence, mobility control was proposed as a main new EOR mechanism for colloidal silica nanoparticle flooding, promoting volumetric sweep efficiency by increasing displacing fluid viscosity. Another important factor, which significantly affects EOR results, is wettability. Herein, reinforced functional colloidal silica nanoparticles in wettability alteration experiments and their effects were inspected on the contact angles. Intermolecular interactions were found to be the underlying mechanism of the wettability alterations. The nanoparticles form a wedge film near where oil, water, and solid surface coexist and scrape away oil film by a pressure gradient originated from their self-assembly on the solid surface (see Fig. 14b). The existence of oleophobic functional groups like Si–OH in silica structure caused wettability transition to mixed-wet and partially water-wet, respectively. This mechanism, by which oil droplets were detached from the rock surface, could lead to more oil production for silica nanoparticles. Nanoparticles have been proposed to be used during EOR operations to accomplish superior results concerning IFT reduction. Adsorption of the nano-sized materials onto the joint surface between oil and water will effectively lessen the IFT and in turn capillary force (see Fig. 14c). This clarified that strongly hydrophilic nanoparticles like silicon oxide are able to activate interfacial phenomena. In conclusion, the reinforced colloidal silica nanoparticles did so through three important EOR mechanisms of mobility control, wettability restoration, and IFT reduction, as illustrated in Fig. 14.

## Conclusions

In this study, colloidal silica nanofluid was synthesized using sodium silicate, and its effect on alteration in interfacial tension of water containing nanofluid and oil, alteration in wettability, secondary and enhanced recovery was assessed. First, colloidal nanofluid containing nano-silica was successfully synthesized by using the sol–gel method. FTIR test, particle size, and zeta potential indicated that silica particles are formed in fluid structure, and average particle size falls in nanoscale, and the solution was stable. Besides, the effect of adding nanofluid to seawater at diluted concentrations of 1, 20, and 40 times was tested in order to assess the effect of salt on the stability of nanoparticles. The results obtained by observation and alteration in particle size indicated that seawater containing nanofluid diluted by 40 times is of favorable stability. In order to study the impact of nanofluid on alteration in interfacial tension, seawater containing nanofluid and oil in stable solutions was tested. The results showed that seawater containing nanofluid diluted by 40 times has the maximum effect on reducing interfacial tension equal to 41%. Afterward, the alteration in wettability was studied. According to the results, the seawater containing nanofluid diluted by 40 times could obtain a 49% alteration in wettability on carbonate core rock samples. According to mechanisms, including reduced interfacial tension and wettability, seawater containing nanofluid diluted by 40 times had the maximum positive effect on reducing interfacial tension and altered in wettability to water-wet was selected for secondary and enhanced recovery. According to core flooding results, the seawater that contained nanofluid improved secondary oil recovery by 13.7%. It also increased enhanced oil recovery by 8.3%, indicating the favorable performance of synthesized nanoparticles in oil recovery mechanisms.

## Data Availability

All data generated or analyzed during this study are included in this published article.
